# Timing of Orthodontic Intervention for Pediatric Class II Malocclusion: A Systematic Review on Early vs. Late Treatment Outcomes

**DOI:** 10.3390/children12111533

**Published:** 2025-11-13

**Authors:** Stefania Dinu, Andreea Igna, Emanuela Lidia Petrescu, Emilia Brandusa Braila, Dorin Cristian Dinu, Razvan Mihai Horhat, Cristina Mihai, Iuliana-Anamaria Traila, Diana Florina Nica, Malina Popa

**Affiliations:** 1 Department of Pediatric Dentistry, Faculty of Dental Medicine, “Victor Babes” University of Medicine and Pharmacy, 9 No., Revolutiei 1989 Bv., 300041 Timisoara, Romania; dinu.stefania@umft.ro (S.D.); igna.andreea@umft.ro (A.I.); popa.malina@umft.ro (M.P.); 2 Pediatric Dentistry Research Center, Faculty of Dental Medicine, “Victor Babes” University of Medicine and Pharmacy, 9 No., Revolutiei 1989 Bv., 300041 Timisoara, Romania; 3 Department of Prosthesis Technology and Dental Materials, “Victor Babes” University of Medicine and Pharmacy, 9 No., Revolutiei 1989 Bv., 300041 Timisoara, Romania; petrescu.emanuela@umft.ro; 4 Dental Research Center Using Conventional and Alternative Technologies, Faculty of Dental Medicine, “Victor Babes” University of Medicine and Pharmacy, 9 No., Revolutiei 1989 Bv., 300041 Timisoara, Romania; 5 Doctoral School, ‘Victor Babes’ University of Medicine and Pharmacy Timisoara, Eftimie Murgu Square 6 No. 2, 300041 Timisoara, Romania; braila.emilia@umft.ro; 6 Family Dental Clinic, Private Practice, 24 Budapesta Street, 307160 Dumbravita, Romania; dorin@dr-dinu.com; 7 Department of Conservative Dentistry and Endodontics, Faculty of Dental Medicine, “Victor Babes” University of Medicine and Pharmacy Timisoara, Eftimie Murgu Square 2, 300041 Timisoara, Romania; horhat.razvan@umft.ro; 8 Advanced and Digital Endodontic, Restorative and Prosthodontic Treatment (TADERP) Research Center, Department of Conservative Dentistry and Endodontics, Faculty of Dental Medicine, “Victor Babes” University of Medicine and Pharmacy Timisoara, Eftimie Murgu Square 2, 300041 Timisoara, Romania; 9 Dentalmed Luxury Dental, Private Practice, 011858 Bucharest, Romania; cristina.mihai.dmd@gmail.com; 10 Department of Microscopic Morphology-Anatomic Pathology, ANAPATMOL Research Center, “Victor Babes” University of Medicine and Pharmacy, 300041 Timisoara, Romania; 11 Department of Anesthesiology and Oral Surgery, School of Dental Medicine, “Victor Babes” University of Medicine and Pharmacy of Timisoara, 2A Eftimie Murgu Place, 300041 Timisoara, Romania; 12 Research Center of Dento-Alveolar Surgery, Anesthesia and Sedation in Dental Medicine, Faculty of Dental Medicine, “Victor Babes” University of Medicine and Pharmacy of Timisoara, 2A Eftimie Murgu Place, 300041 Timisoara, Romania

**Keywords:** early orthodontic treatment, class II malocclusion, growth modification, treatment timing, pediatric patients, cervical headgear

## Abstract

**Highlights:**

**What are the main findings?**

- Early orthodontic intervention significantly improves skeletal development, arch dimensions, and airway space in patients with Class II malocclusion.
- Statistically significant differences favor early treatment in parameters such as gonial angle, maxillary width, and dental arch length.

**What are the implications of the main finding?**

- Timely application of growth-modifying appliances enhances treatment outcomes and may reduce the need for extractions or prolonged fixed appliance therapy.
- Individualized treatment timing, particularly favoring early intervention in selected cases, should be integrated into evidence-based planning for orthodontic treatment in pediatric patients.

**Abstract:**

**Background/Objectives:** The optimal timing for orthodontic treatment in pediatric patients with malocclusion, particularly Class II discrepancies, remains a topic of ongoing clinical debate. Early treatment during the mixed dentition stage harnesses craniofacial growth potential, whereas later intervention may capitalize on pubertal growth for greater skeletal correction, especially for skeletal and airway improvements. This systematic review aimed to compare the outcomes of early versus late orthodontic treatment to assess their relative effectiveness. **Methods:** A systematic review was conducted in accordance with PRISMA guidelines, including randomized controlled trials and observational studies published between 2015 and 2025. Eleven studies comparing early and late treatment were analyzed, and the risk of bias was evaluated using standardized assessment tools. **Results:** Of the eleven studies, eight reported statistically significant improvements favoring early orthodontic intervention. Early treatment was associated with greater enhancement of maxillary and mandibular arch development, improved jaw relationships, and expanded airway dimensions. Studies utilizing headgear or other growth-modifying appliances also showed more favorable eruption patterns and alignment, underscoring the clinical relevance of early-phase management. **Conclusions:** Early orthodontic treatment can provide meaningful benefits in guiding skeletal growth, improving dental arch form, and enhancing treatment efficiency. These benefits were most consistently supported in skeletal and airway outcome domains. While late treatment may be suitable for some cases, personalized planning remains essential. Further large-scale, standardized longitudinal studies are needed to refine treatment-timing protocols in pediatric orthodontics.

## 1. Introduction

The optimal timing of orthodontic intervention in pediatric patients with malocclusion remains a topic of ongoing debate. Proponents of early treatment advocate initiating therapy during the deciduous or early mixed dentition (typically between 6 and 9 years of age) to guide craniofacial growth and prevent worsening of skeletal discrepancies. In contrast, advocates of later intervention recommend treatment during the pubertal growth spurt (approximately 11 to 13 years of age), when peak mandibular growth can be harnessed for more efficient skeletal correction and improved treatment stability [1,2].

Malocclusion, encompassing both dental misalignments and skeletal discrepancies, is among the most prevalent developmental conditions in children, with potential functional, aesthetic, and psychosocial consequences if left untreated. Prevalence rates range from 20% to 93% depending on study design and age group [3]. It can lead to issues such as incisor trauma, impaired oral hygiene, bullying, and reduced quality of life [1,2].

Early orthodontic intervention, often referred to as interceptive treatment, is typically initiated during the early mixed dentition phase and addresses skeletal or dental discrepancies, such as Class II or III malocclusions, posterior crossbites, or significant arch length discrepancies. Studies suggest early intervention may help reduce overjet-related incisor trauma, improve psychosocial well-being, and simplify later treatment phases. For instance, an increased overjet (>5 mm) in children aged 7 to 14 is associated with more than double the risk of dental trauma, reinforcing the argument for early intervention in selected cases [1,2].

However, early treatment often results primarily in dentoalveolar instead of actual skeletal changes, and skeletal effects may relapse unless reinforced during the adolescent growth phase. For Class II malocclusion, functional appliance therapy in the prepubertal stage yields modest benefits and frequently requires a second treatment phase during puberty. In contrast, treatment initiated during the pubertal growth spurt results in greater and more stable mandibular advancement [4,5]. Although the American Association of Orthodontists recommends an initial screening by age 7, evidence supporting routine early treatment remains limited. Excessive use of early orthodontics may increase treatment duration, costs, and patient fatigue or loss of motivation without long-term benefit if patient selection is inappropriate [2,6].

The central dilemma in pediatric orthodontics is determining the optimal timing for intervention—early (approximately 6–9 years) or late (around 11–14 years of age). Early (interceptive) treatment leverages growth potential to correct developing anomalies and is often indicated for posterior crossbites, skeletal Class III malocclusions, or pronounced arch length discrepancies. Late treatment during puberty may be more effective for skeletal discrepancies such as mandibular retrusion and is typically better tolerated by older and more compliant patients. Ultimately, timing should be individualized based on growth pattern, malocclusion severity, and psychosocial factors [4,6,7].

Despite decades of clinical research, the optimal timing of orthodontic intervention in pediatric patients continues to be a subject of professional debate. Given this clinical uncertainty and the significant implications of treatment timing on outcomes, burden of care, and resource allocation, a systematic synthesis of the available evidence is both necessary and timely.

This systematic review aims to address the following research questions (PICO format):

- Population (P): children (6–14 years old) with malocclusion, primarily Class II cases.
- Intervention (I): early orthodontic treatment initiated during the deciduous or early mixed dentition phase (evaluate the effectiveness of growth-modifying appliances (e.g., cervical headgear (CH), Modified C-palatal plates (MCPP), the Eruption Guidance Appliance, EGA) when applied at different stages of dentofacial development).
- Comparison (C): late orthodontic treatment initiated during the late mixed or early permanent dentition phase.
- Outcomes (O): Skeletal, dental, and airway-related changes; treatment duration and efficiency; need for extractions or fixed appliances; and long-term stability.

By addressing these questions, the review seeks to provide evidence-based guidance for clinicians on optimizing treatment timing and improving individualized, growth-sensitive orthodontic care for pediatric patients.

## 2. Materials and Methods

### 2.1. Study Design

This article is a systematic review conducted in accordance with the PRISMA 2020 (Preferred Reporting Items for Systematic Reviews and Meta-Analyses, see File S1) guidelines [8] and registered in the International Prospective Register of Systematic Reviews (PROSPERO) under the registration number CRD420251157567. Its purpose is to synthesize and critically appraise current evidence comparing early versus late orthodontic treatment in children with malocclusion. The review was designed to answer a focused clinical question (*In children with Class II malocclusion, does early orthodontic intervention lead to better skeletal and dental outcomes compared to treatment started later during puberty?*) using predefined inclusion criteria, databases, and methodological steps. It aimed to provide clarity on the clinical effectiveness, treatment duration, stability, and psychosocial impacts associated with the timing of orthodontic intervention.

Elicit AI was used exclusively to organize preliminary literature during title and abstract screening, whereas ChatGPT (GPT-4-turbo, April 2025) was employed to refine the language of the author-written text. Neither tool was used for study selection, data extraction, analysis, or interpretation. All methodological decisions and evaluations were performed manually by the authors. All authors verified the accuracy and integrity of the content.

### 2.2. Study Selection and Eligibility Criteria

To ensure methodological rigor and clinical relevance, studies included in this systematic review were selected through a structured, multi-phase screening process: first with Elicit AI and then rigorously reviewed by the authors. The initial search across multiple databases (Semantic Scholar and PubMed) identified 496 potentially relevant articles published over the past decade (2015–2025). Gray literature and clinical trial registries were not systematically searched, as the review focused on peer-reviewed journal articles indexed in major databases. These were first screened by title and abstract, followed by full-text eligibility assessments. Only studies that directly compared early versus late orthodontic interventions in pediatric patients with malocclusion were included in the final analysis.

Eligible studies met several predefined criteria:

1. The population of interest included children aged 6 to 14 years who were free from craniofacial syndromes or cleft conditions.
2. The intervention had to involve orthodontic treatment with fixed, removable, or functional appliances, initiated either in early childhood (ages 6–9) or during the later mixed to early permanent dentition stage (ages 10–14).
3. Essentially, studies were required to offer a clear comparison between early and late treatment groups, ideally with at least a two-year age gap between cohorts.
4. Only studies published in English were included.
5. Included studies also had to report on at least one of the following outcomes: occlusal changes, treatment duration, post-treatment stability, or patient-centered outcomes such as satisfaction or psychosocial impact.

Exclusion criteria included the following (Excluded studies can be seen in Supplementary Materials Table S1):

1. Case reports, narrative or systematic reviews, editorials, and expert opinions.
2. Studies involving non-orthodontic interventions or mixed-age samples without distinct early and late treatment groups.
3. Articles lacking sufficient data for extraction or reporting outcomes unrelated to treatment timing.

**Figure 1:**
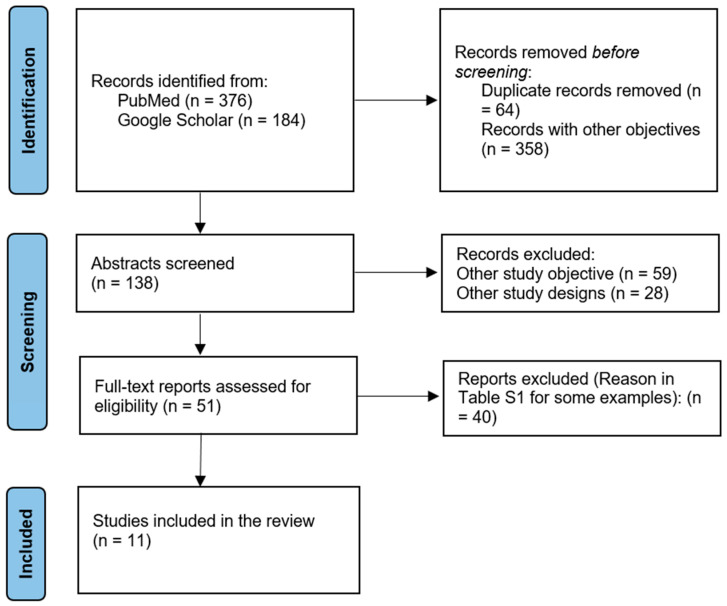
PRISMA flow diagram.

Only studies employing robust research designs were considered, including randomized controlled trials, controlled clinical trials, prospective cohorts, and retrospective studies. Additionally, a follow-up period was required after treatment to evaluate the long-term effects. A total of 11 studies met these inclusion criteria, and their data were extracted systematically for qualitative synthesis. The study selection process is summarized in the PRISMA flow diagram (Figure 1).

### 2.3. Data Items and Data Analysis

From each included study, relevant data were systematically extracted using a predefined data extraction form. The primary data items included: study identification (first author, year of publication), country of origin, study design, age range or mean age of participants, sample size, and type of malocclusion treated. Information was also gathered on the orthodontic treatment modality used and the specific outcomes assessed.

To assess treatment timing, the studies were categorized based on whether interventions began in early childhood (typically 6–9 years of age) or in later stages of development (10–14 years). Follow-up durations were recorded to distinguish between short- and long-term effects, including post-retention outcomes when available. Measurement methods varied among studies and included lateral cephalograms, 3D model analyses, dental casts, panoramic radiographs, and patient questionnaires.

Heterogeneity among studies was quantitatively assessed (I^2^ > 75%) and was considered indicative of substantial heterogeneity, confirming significant variability in outcome measures, statistical methods, and follow-up durations. Due to this high heterogeneity and lack of standardized outcome variables, a quantitative meta-analysis was deemed inappropriate, and a qualitative synthesis was performed instead. When available, effect sizes, *p*-values, and confidence intervals were used to interpret the strength and significance of the results. Particular attention was paid to differences in treatment effectiveness, eruption timing, arch development, cost efficiency, and gender-specific responses to early treatment. The findings were synthesized to provide a comparative overview of how treatment timing influences clinical outcomes in pediatric malocclusion.

All data were organized and analyzed using Python version 3.12. Given the nature of the dataset, the analysis employed both descriptive and comparative statistical techniques. Descriptive statistics summarized baseline characteristics and outcomes across early and late treatment groups.

For non-parametric comparisons, a composite ‘success score’ was calculated for each study, reflecting the proportion of reported favorable clinical outcomes relative to total evaluated parameters. This standardized measure enabled cross-study comparisons while accounting for differences in outcome scales. For between-group comparisons, independent-sample t-tests or the Mann–Whitney U test were used, depending on data normality. Statistical significance was defined as *p* < 0.05.

### 2.4. Risk of Bias Assessment

The risk of bias for the included studies was assessed using the RoB 2.0 tool across five domains: randomization process, deviations from intended interventions, missing outcome data, measurement of the outcome, and selective reporting (Table S2 in Supplementary Materials). Two independent reviewers assessed each study’s risk of bias. Any disagreements were discussed and resolved by consensus; when consensus could not be reached, a third reviewer adjudicated. Overall, the methodological quality of most studies was high, with 8 of the 11 studies assessed as having a low risk of bias across all domains.

## 3. Results

### 3.1. Overview of Selected Studies

To synthesize the findings of the eleven included studies, we analyzed and compared the data across key findings (Table 1 and Table S3 in the Supplementary Materials).

Most included studies were randomized controlled trials (RCTs), which ensured high internal validity. Specifically, studies by Julku et al. (2019, both entries), Käsmä et al. (2025), Kallunki et al. (2022), Kim et al. (2024), Mandall et al. (2022), Hannula et al. (2023), and Julku et al. (2018) [9,10,11,12,13,14,15,16] were RCTs conducted primarily in Finland, Korea, Sweden, and the UK. Männchen et al. (2022) and Fourneron et al. (2020) employed retrospective designs [17,18], while Myrlund et al. (2018) was a prospective cohort study [19]. This spread provided both experimental rigor and real-world relevance.

**Table 1:** Study characteristics.

Study ID	Country	Study Design	Age Range/ Mean Age (years)	Number of Participants	Type of Malocclusion	Treatment Modality	Follow-Up Duration	Skeletal Maturity Stage at Treatment Onset	Key Findings
Julku et al., 2019 [9]	Finland	RCT	7–18	56	Class II	Cervical headgear (CH)	11 yrs	EG = Prepubertal (CVMS I–II); LG = Pubertal (CVMS III)	Early treatment improved the anteroposterior jaw relationship, with minimal differences in long-term outcomes.
Käsmä et al., 2025 [12]	Finland	RCT	7–18	67	Class II	CH treatment	14 yrs	EG = Prepubertal (CVMS I–II); LG = Pubertal (CVMS III)	Later treatment improved eruption timing and alignment; the early group showed more molar tipping.
Kallunki et al., 2022 [13]	Sweden	RCT	9–11	56	Class II	Headgear activator	24 mo	EG = Prepubertal (CVMS I–II); LG = Pubertal (CVMS III)	Similar costs and outcomes were observed between the early and late groups, with a reduction in overjet in both.
Julku et al., 2019 [10]	Finland	RCT	Mean age 7.2	67	Class II	CH treatment	24–26 mo	EG = Prepubertal (CVMS I–II); LG = Pubertal (CVMS III)	Early CH treatment is more effective in males due to differences in skeletal maturation.
Kim et al., 2024 [14]	Korea	RCT	9.6–12.3	71	Hyperdivergent Class II	MCPP and CH	-	EG = Pubertal (CVMS III); LG = Postpubertal (CVMS IV)	Early MCPP showed greater vertical control and skeletal balance (*p* < 0.010).
Männchen et al., 2022 [17]	Italy	Retrospective study	8–18	527	Class II	Functional and fixed appliances	6 yrs	Not reported	Early treatment reduced the need for extraction and fixed appliances, but resulted in a longer total treatment time.
Mandall et al., 2022 [15]	United Kingdom	RCT	11–13	75	Class II	Rapid maxillary expansion and facemask	≥3 yrs	Not reported	Early intervention reduced ANB and overjet; similar psychosocial outcomes.
Julku et al., 2018 [11]	Finland	RCT	7–11.5	67	Class II	CH treatment	4.5 yrs	Prepubertal (CVMS I–II)	Significant posterior maxillary movement in early treatment males.
Myrlund et al., 2018 [19]	Norway	Prospective cohort study	7.7–9.1	35	Class II	EGA	6 yrs	EG = Prepubertal (CVMS I–II); LG = Pubertal (CVMS III)	Significant improvements in overjet, overbite, and crowding after early intervention.
Fourneron et al., 2020 [18]	France	Retrospective study	<7 vs. ≤13	40	Unilateral posterior crossbite (UPCB)	Quad Helix (QH)	18 mo	EG = Prepubertal (CVMS I–II); LG = Pubertal–Postpubertal (CVMS III–IV)	Early treatment improved mandibular asymmetry correction (+1 mm, *p* = 0.008)
Hannula et al., 2023 [16]	Finland	RCT	7–18	46	Class II	CH treatment	14 yrs	Prepubertal (CVMS I–II)	The early group showed greater gains in arch width and length, particularly in males.

RCT = randomized controlled trial; CH = cervical headgear; MCPP = modified C-palatal plate; EG = early group; LG = late group.

Age ranges varied widely. The youngest participants were under 7 years in Fourneron et al. (2020), while the oldest extended to 18 years in Käsmä et al. (2025) and Hannula et al. (2023) [12,16,18]. Sample sizes ranged from as few as 35 (Myrlund et al., 2018) to as many as 527 (Männchen et al., 2022), illustrating the variability in statistical power among studies [17,19]. Most studies stratified by age or grouped subjects into early (EG) and late treatment (LG) cohorts.

Class II malocclusion was the predominant condition studied, appearing in at least ten of the eleven studies (e.g., Julku et al., Käsmä et al., Kim et al., Hannula et al.) [11,12,14,16,17]. Other conditions included hyperdivergent Class II (Kim et al., 2024), unilateral posterior crossbite (Fourneron et al., 2020), and mandibular crowding (Myrlund et al., 2018) [14,18,19]. This uniformity reinforced the relevance of findings to Class II treatment timing.

Interventions varied but commonly included CH and other growth-modifying appliances. Julku et al., Käsmä et al., and Hannula et al. used Kloehn-type CH [9,10,11,12,16]; Kim et al. applied MCPP and CH [14]; Männchen et al. used a broad mix of appliances [17]; Myrlund et al. employed EGA [19]; and Fourneron used Quad Helix for crossbite correction [18]. While CH remained dominant, the studies provided a comparative view of the diverse effects of various appliances.

Outcomes were both skeletal and dental. Many studies focused on cephalometric changes (e.g., SNA, Sella–nasion to gonion–gnathion angle (SN-GoGn), Frankfort–mandibular plane angle (FMA), N-ANS, gonial angle), dental arch dimensions, overjet and overbite, molar relations, and airway parameters. Hannula et al. and Julku et al. used 3D scanning for arch analysis [9,10,11,16], whereas Kim et al. emphasized vertical control measures. Männchen et al. uniquely addressed extraction rates and treatment burden [17].

Follow-up ranged from 2 years (Kallunki et al., 2022) to 14 years (Käsmä et al., 2025; Hannula et al., 2023) [12,13,16]. Several studies (e.g., Julku et al., 2019; Hannula et al., 2023) reported long-term post-retention results, which add weight to their conclusions about treatment stability [9,16].

There was no uniform superiority of early or late treatment. Julku et al. (2019) and Käsmä et al. (2025) found statistically significant but clinically marginal differences [9,12]. Kim et al. (2024) demonstrated superior vertical control with early MCPP [14]. Männchen et al. (2022) demonstrated practical advantages of early treatment in reducing the need for extractions [17]. Hannula et al. (2023) and Julku et al. (2018) highlighted sex-specific skeletal responses that favor early intervention [11,16]. Meanwhile, Kallunki et al. and Mandall et al. suggested similar outcomes, regardless of timing, although early treatment may require longer active phases [13,15].

Eleven studies were included, encompassing 749 patients. The average age at the start of treatment across the studies was 9.43 years, while the mean age at the end of follow-up (for studies reporting it) was 14.58 years. Age ranges varied widely, with the youngest participants starting treatment at 7 years old and the oldest participants followed up to 18 years old.

The average follow-up duration after the initial intervention was approximately 4.34 years, although this varied considerably across studies and treatment protocols. Longitudinal studies like those of Käsmä et al. (2025) and Hannula et al. (2023) provided extended follow-up durations of over 6 and 10 years, respectively, offering insights into long-term treatment effects and stability [12,16].

Regarding treatment modalities, the most frequently applied approach was CH, used in 5 of the 11 studies. Other modalities included headgear activator combinations, MCPP, rapid maxillary expansion (RME) with facemask (FM), EGA, and Quad Helix devices. One study by Männchen et al. (2022) employed a mixed design, incorporating both early functional and late fixed appliances, reflecting the real-world clinical flexibility [17].

The observed heterogeneity in treatment approaches and timing illustrates the complexity of determining an optimal intervention strategy for pediatric malocclusion. The data suggest that while early treatment is standard, especially for skeletal Class II cases, later interventions remain a viable and sometimes preferable option, depending on the growth stage, appliance type, and severity of malocclusion.

### 3.2. Risk of Bias

All studies included in this systematic review were assessed using the revised Cochrane Risk of Bias 2.0 (RoB 2.0) across five domains: randomization, deviations from intended interventions, missing outcome data, measurement of outcomes, and selective reporting (Table S2, available in the Supplementary Materials). The overall quality of the evidence was considered acceptable, with the majority of studies demonstrating a low risk of bias across all domains.

Out of the 11 studies analyzed, seven were judged to have a low overall risk of bias, including all studies by Julku et al. (2018, 2019), Käsmä et al. (2025), Mandall et al. (2022), Myrlund et al. (2018), and Hannula et al. (2023) [9,10,11,12,15,16,19]. These studies employed robust methodologies, appropriate randomization procedures, and reliable outcome measurements, with minimal issues related to missing or selectively reported data.

Some concerns were identified in four studies. Kallunki et al. (2022) and Kim et al. (2024) showed uncertainties in their randomization procedures and potential deviations from the intended interventions [13,14], although their outcome reporting and data completeness remained strong. Männchen et al. (2022) [17], a retrospective study, was classified as having a moderate overall risk of bias, primarily due to concerns about missing outcome data and moderate risks of outcome measurement bias and selective reporting, which are inherent limitations of its non-randomized design. Similarly, Fourneron et al. (2020) raised some concerns related to the randomization process, although it otherwise maintained a low risk across other domains [18].

### 3.3. Statistical Analysis

Both descriptive and inferential statistical analyses were conducted to compare baseline characteristics and follow-up durations between studies implementing early and late orthodontic interventions (Supplementary Materials, Table S4). Studies categorized under both early and late treatment protocols were included in both group comparisons to reflect the full scope of dual-phase treatment assessments.

**Figure 2:**
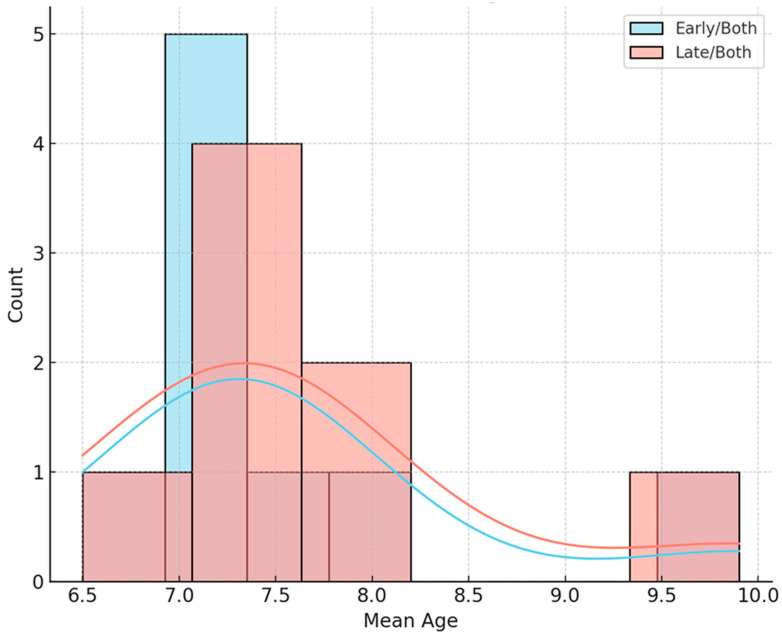
Distribution of mean age at start.

The analysis of mean age at treatment initiation revealed comparable distributions between the early and late groups, as well as between both groups. The mean starting age in early-treatment studies was similar to that in late-treatment studies. An independent-samples t-test revealed no statistically significant difference in mean age at treatment onset between groups (t = −0.10, *p* = 0.920), indicating that patient inclusion criteria regarding age were relatively consistent across studies, regardless of treatment timing (Figure 2).

**Figure 3:**
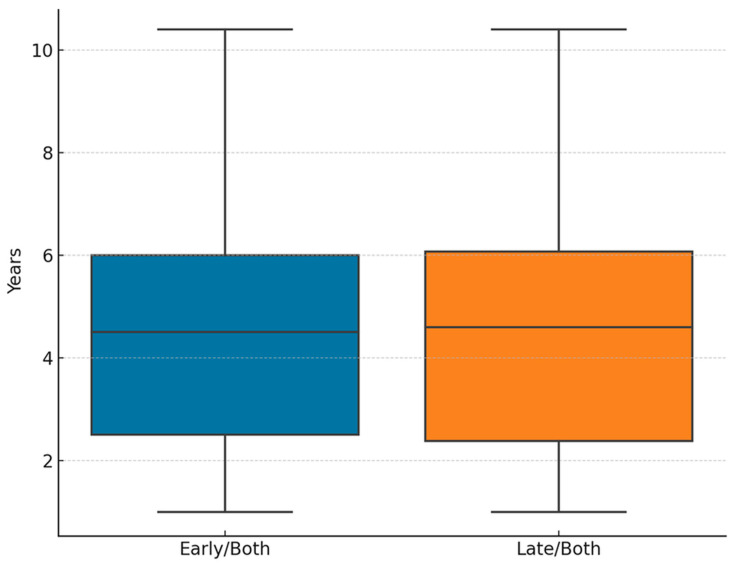
Follow-up duration comparison.

Similarly, the duration of follow-up did not differ significantly between groups. The mean follow-up duration for the early and late treatment studies was nearly equivalent to that for the late/both studies, as supported by the *t*-test result (t = −0.01, *p* = 0.990) (Figure 3). This consistency reinforced the comparability of outcome observation periods, an essential aspect when synthesizing longitudinal data. These results indicate methodological homogeneity among the selected studies in terms of baseline age and observational duration, thereby enhancing the robustness of subsequent clinical comparisons between early and late interventions.

To further investigate whether the complexity of orthodontic treatment protocols influences clinical outcomes, we compared estimated success scores for single-modality treatments with those for mixed or step-by-step interventions. Single-modality treatments refer to protocols that use one primary device (e.g., CH, quad helix), whereas mixed protocols include combinations such as functional appliances with fixed devices or space maintainers. The analysis revealed that single-modality treatments achieved a mean success score of 2.75 (±0.46), compared with 2.33 (±0.58) for mixed-treatment protocols. Although the difference was not statistically tested due to limited sample size, the descriptive trend suggested that simpler, well-targeted interventions could be as effective or more efficient than complex multi-appliance sequences.

This pattern may have reflected improved compliance with simpler devices, shorter active treatment periods, and better alignment with growth modification strategies. However, mixed protocols might still have offered specific advantages in challenging or multidimensional cases, even if the overall effectiveness appeared diluted across broader indications. These findings emphasize the need to balance treatment complexity with clinical practicality, particularly when considering early interventions in pediatric patients.

To statistically validate the observed difference in treatment outcomes between single and mixed-modality interventions, a Mann–Whitney U test was performed. This non-parametric test is appropriate given the ordinal nature and small sample size of the success ratings. The results showed no statistically significant difference between the two groups (U = 17.0, *p* = 0.270), suggesting that the observed variation in mean success scores, although present, was not significant enough to reject the null hypothesis under standard significance levels.

However, the effect size (r = 0.31; 95%CI 0.05–0.54) indicated a small to moderate practical difference between the treatment complexities. This effect size, calculated from the Mann–Whitney U z-score, indicates that single-modality interventions tended to produce better estimated clinical outcomes, even though the sample size limited statistical power (Table 2).

**Figure 4:**
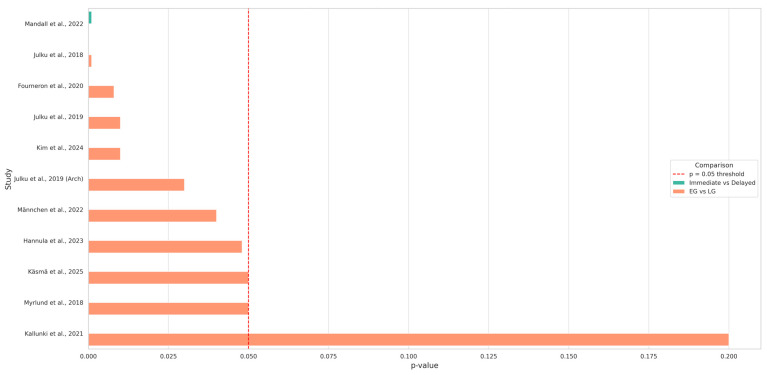
Statistical significance of key findings across studies (early vs. late treatment) (Data from [9,10,11,12,13,14,15,16,17,18,19].

The statistical analysis of the key findings across the included studies revealed a consistent pattern favoring early treatment interventions in multiple domains. As visualized in the bar plot, the majority of studies reported statistically significant differences (*p* < 0.050) between EG and LG treatment groups (Figure 4).

For instance, Julku et al. (2019) and Julku et al. (2018) showed significant skeletal and airway improvements in the early treatment group, with *p*-values of 0.01 and 0.001, respectively [9,11]. Similarly, Hannula et al. (2023) reported multiple dental arch improvements in the EG, including intermolar and intercanine widths, with highly significant *p*-values (e.g., *p* = 0.001 for upper canine width) [16].

Studies such as Käsmä et al. (2025) and Männchen et al. (2022) also demonstrated significant effects in eruption timing and reduction in treatment complexity, respectively [12,17]. Mandall et al. (2022) observed notable improvements in the ANB angle and a decrease in overjet in the immediate treatment group (*p* < 0.001) [15]. In contrast, Kallunki et al. (2022), which focused on cost analysis and quality-of-life metrics, did not find statistically significant group differences (*p* = 0.200), indicating that not all outcomes favored early intervention [13] (Table 3).

Comparing the studies that favored EG versus those that favored LG (Mann–Whitney U test) yielded a statistically significant result (U = 8.0, *p* = 0.006). This suggests that early treatment is more frequently associated with favorable outcomes than late treatment across the reviewed studies.

## 4. Discussion

Of the eleven studies included in this review, eight reported statistically significant advantages of early orthodontic intervention, mainly related to skeletal growth, maxillary expansion, and airway enhancement. The overall pattern, supported by the Mann–Whitney U test (U = 8.0, *p* = 0.006), indicates that early treatment generally provides more favorable outcomes than treatment initiated later. Although some studies, such as Käsmä et al. (2025), reported comparable or slightly better dental alignment with later treatment, most evidence supports the clinical relevance of timely early intervention [12]. Effect size analyses further suggest moderate benefits for simpler, growth-sensitive protocols when applied during active developmental phases.

These findings align with the broader literature evaluating optimal treatment timing for Class II malocclusion. Recent high-level evidence indicates that early treatment offers limited long-term advantages over one-phase treatment in adolescence [20,21]. Although outcomes are similar, early intervention does produce notable short-term changes. Starting Class II correction in the mixed dentition (ages 7–10) can significantly reduce an excessive overjet and improve molar relationships during those early years [20,22,23]. A recent 2024 analysis reported greater interim improvements in molar relationship and incisor position with early treatment (mean molar correction ~4.5 mm early vs. 3.1 mm late) [22]. Furthermore, early Class II correction “works” in the short term by correcting the bite approximately 1–2 years earlier. However, studies have found no sustained superiority of the final result over one-phase treatment. These findings partially corroborate the present results.

Nevertheless, specific clinical scenarios may justify initiating treatment at a later stage. For instance, in patients who have completed most of their growth or in those with limited compliance during early childhood, postponing intervention can improve cooperation and overall treatment predictability. Additionally, late treatment may be preferable when comprehensive orthodontic correction requires full permanent dentition or when skeletal growth modification is no longer feasible [21].

A primary goal of early Class II treatment is to modify growth and promote favorable jaw development. Functional appliances, such as the Twin Block or Herbst, aim to stimulate mandibular growth in retrognathic Class II patients, whereas headgear aims to restrain forward maxillary growth [24]. Recent reviews confirm that the early use of functional appliances produces measurable skeletal changes in the short term. There is high-level evidence that early functional-appliance therapy can enhance mandibular position and length and reduce a Class II jaw discrepancy by a small but significant amount [25]. For example, a systematic review (2018) found that early functional treatment improved sagittal skeletal relationships primarily by advancing the mandible forward [26]. Additionally, skeletal corrections from early treatment are clinically real but not exclusive to early intervention; proper timing around the pubertal peak can achieve similar jaw corrections in a single phase [27].

Regarding the appliances used in practice, the Twin Block (removable) and Herbst (fixed) are prominent growth-modification appliances for Class II correction. These appliances posture the mandible forward and are typically used during growth to stimulate mandibular development [28,29]. Studies from 2024 onward have consistently shown that both Twin Blocks and Herbst appliances effectively correct Class II malocclusion, primarily through dentoalveolar changes with a modest skeletal contribution [30]. A key difference lies in compliance and efficiency. A new 2023 randomized trial comparing removable Twin Block versus fixed Herbst appliances (Hanks Herbst) found both appliances corrected overjet, but the Herbst did so more quickly and reliably. Patients with Herbst achieved normal overjet approximately 1.5 months faster on average, with a higher success rate (only about 17% of cases were unsuccessful compared to 37% in the Twin Block group, which never achieved full correction). Herbst’s fixed nature ensured cooperation, whereas Twin Block outcomes were more variable due to compliance issues with wear time. Total treatment time in that trial was ~9 months for Herbst vs. ~10 months for Twin Block (for the overjet reduction phase). The downside was that Herbst patients required more chairside time and experienced more appliance breakages; however, overall, no significant differences in patient-reported quality of life were noted. The authors concluded that Herbst is more efficient and predictable in reducing Class II overjet, though both appliances ultimately produced similar occlusal outcomes [31,32].

Cervical pull headgear, such as the Kloehn-type, is a traditional Phase I appliance used to correct Class II malocclusion by restricting maxillary growth and distalizing upper molars [33,34]. Applied mainly during the late mixed dentition, it reduces overjet and can decrease ANB by limiting forward maxillary displacement. Early use may correct Class II discrepancies sooner, although later treatment can achieve comparable results using alternative approaches. Success depends heavily on compliance, as 10–14 h of daily wear are typically required. When worn effectively, headgear provides orthopedic restraint of the maxilla, reducing Class II severity until compensatory mandibular growth occurs [17,35,36,37]. The present review indicates that treatment outcomes vary with timing, but appliances applied in harmony with individual growth stages yield the most favorable outcomes.

Two modern methods for early Class II treatment are the MCPP and the EGA, each offering specific clinical benefits. The MCPP yields skeletal and dental results similar to traditional headgear, with better vertical control through maxillary molar intrusion and a lower mandibular plane angle [38,39,40]. In early mixed-dentition patients treated with the EGA, the occurrence of Class II decreased from 100% to 14%, with notable improvements in mandibular length and a lower ANB compared to controls [41,42]. A 2022 review confirmed EGA’s effectiveness in reducing overjet and overbite, though long-term controlled studies are still needed [43]. Overall, these elastodontic devices are effective and biocompatible across various orthodontic applications [44]. This study shows that the MCPP provided better vertical control in hyperdivergent Class II cases. The early MCPP group experienced a 0.6° reduction in the SN-GoGn angle. Conversely, the late MCPP and headgear groups saw this angle increase, suggesting that using MCPP at the right time is effective in managing vertical growth. Additionally, the EGA used in early mixed dentition produced significant improvements in overjet, overbite, and mandibular crowding, highlighting its role in functional dentoalveolar remodeling.

This supports the clinical observation that targeted interventions, such as cervical headgear, can produce highly effective results with less complexity. Meanwhile, mixed-treatment approaches should be reserved for select cases that require multidimensional correction.

However, findings from this study should be interpreted with caution, as outcome heterogeneity, differences in appliance types, and varying follow-up durations limit the ability to make a universal “superiority” claim for early treatment.

The results of this systematic review and comparative statistical analysis highlight the potential benefits of early orthodontic intervention in managing Class II malocclusion, particularly when timed to coincide with periods of active craniofacial growth. However, the heterogeneity in treatment protocols, outcome measures, and follow-up durations across studies highlights the need for future research to adopt standardized methodologies and longitudinal designs. Upcoming investigations should prioritize large-scale, multi-center RCTs that not only compare early versus late intervention but also evaluate the efficacy of specific appliances (CH, MCPP, EGA) across various skeletal patterns and age groups. Moreover, integrating 3D imaging, artificial intelligence-driven growth prediction, and patient-centered outcomes such as quality of life and treatment burden will enhance clinical relevance and inform personalized treatment timing. Exploring the cost-effectiveness and psychosocial impacts of early versus late intervention is an essential dimension for future research.

Despite the comprehensive nature of this review, several limitations must be acknowledged. First, the variable definitions of “early” and “late” treatment across studies, along with differences in outcome measures, appliance protocols, and assessment tools, limit external validity and hinder direct meta-analytic pooling. Second, the relatively small sample sizes and heterogeneity among study populations may introduce bias and restrict the generalizability of the findings. Third, although efforts were made to include both skeletal and dental Class II cases, most high-quality studies focused on specific subtypes or gender-based analyses. Additionally, while the RoB 2.0 assessment indicated low risk of bias in most studies, moderate concerns remained regarding missing outcome data and selective reporting. Not all studies provided effect sizes or confidence intervals, which reduces the ability to uniformly quantify clinical significance. Additionally, heterogeneity in cephalometric measurement techniques—particularly differences between two-dimensional (2D) and three-dimensional (3D) imaging—may influence the comparability of skeletal outcomes across studies. Finally, the partial use of AI-based tools (Elicit AI for literature organization) represents a methodological limitation. However, these tools did not influence data extraction or analysis; their involvement is acknowledged for transparency. Overall, while statistical comparisons suggest trends favoring early intervention, the observational nature of some studies precludes definitive causal inference.

## 5. Conclusions

This systematic review demonstrates that early orthodontic intervention, especially when timed with craniofacial growth phases, can provide measurable short-term benefits in skeletal development, dental arch expansion, and airway improvement in pediatric Class II malocclusion. Early intervention may also reduce the need for extractions or complex multi-phase treatment. However, due to differences in study designs, varying definitions of treatment timing, and limited pooled data, current evidence remains insufficient to establish recommendations on the optimal age or stage for treatment. However, late intervention remains appropriate in non-growing patients, in cases requiring comprehensive orthodontic correction, or when early compliance is limited. Future research should focus on standardized outcome measures, well-powered long-term studies, and quantitative meta-analyses to strengthen the evidence for timing decisions in pediatric orthodontics.

## Figures and Tables

**Table 2:** Descriptive statistics for key variables across early vs. late treatment groups.

Variable	Early/Both Mean ± SD	Late/Both Mean ± SD	t/U Value	*p*-Value	Interpretation
Mean age at treatment start (years)	9.43 ± 1.21	9.51 ± 1.34	t = −0.10	0.920	No significant difference
Mean follow-up duration (years)	4.34 ± 2.76	4.36 ± 2.45	t = −0.01	0.990	Comparable observation length
Composite success score	2.75 ± 0.46	2.33 ± 0.58	U = 17.0	0.270	Not statistically significant
Treatment complexity (single vs. mixed)	—	—	r = 0.31	—	Small to moderate effect favoring single-modality

**Table 3:** Summary of statistically significant findings reported across included studies.

Study	Parameter(s) with Significant Difference	Direction of Effect	*p*-Value	Effect Size/ Magnitude	Favored Group
Julku et al., 2019 [10]	N-ANS, gonial angle	↓ gonial angle, ↑ facial height	0.010	—	Early
Julku et al., 2018 [11]	Airway (rl1–rl2), SNA	↑ airway, ↓ SNA	0.001–0.012	—	Early
Käsmä et al., 2025 [12]	Eruption timing, molar overlap	Better alignment	<0.050	—	Late
Kim et al., 2024 [14]	SN–GoGn, FMA	Improved vertical control	<0.010	Moderate	Early
Hannula et al., 2023 [16]	Intermolar/intercanine width	↑ maxillary width	0.001–0.048	—	Early
Mandall et al., 2022 [15]	ANB, overjet	Reduction in both	<0.001	Large	Early
Männchen et al., 2022 [17]	Extraction rate, FFA use	↓ extractions, ↓ FFA needs	<0.050	—	Early
Fourneron et al., 2020 [18]	Corpus asymmetry (ΔL)	+1.0 mm correction	0.008	—	Early
Kallunki et al., 2022 [13]	Cost, trauma incidence	n.s.	0.200	—	None
Myrlund et al., 2018 [19]	Overjet, overbite, crowding	All improved	<0.050	—	Early

FFA—full fixed appliance; n.s.—not significant.

## Data Availability

Not applicable.

## Supplementary Materials

The following supporting information can be downloaded at: https://www.mdpi.com/article/10.3390/children12111533/s1, File S1. PRISMA 2020 Checklist. Table S1. Excluded Studies and Reason for Exclusion; Table S2. RoB 2.0 Risk of Bias Assessment; Table S3. Study characteristics (extended); Table S4. Extracted data for analysis.

## Abbreviations

The following abbreviations are used in this manuscript:

CH	Cervical Headgear
MCPP	Modified C-palatal Plate
EGA	Eruption Guidance Appliance
RCT	Randomized Controlled Trial
OSA	Obstructive Sleep Apnea
EG	Early Group
LG	Late Group
FFA	Full Fixed Appliance
DPT	Dental Panoramic Tomograph
ANB	A point–Nasion–B point (cephalometric angle)
FMA	Frankfort–Mandibular Plane Angle
SN-GoGn	Sella–Nasion to Gonion–Gnathion angle
NSL-PL	Nasion–Sella Line to Palatal Line
rl1–rl2	Retroglottic airway linear measurement
