# Supplementary material for: Timing of Orthodontic Intervention for Pediatric Class II Malocclusion: A Systematic Review on Early vs. Late Treatment Outcomes
Source: Children (Basel). 2025 Nov 13;12(11):1533. doi: 10.3390/children12111533 (PMC12651552; doi:10.3390/children12111533)
Supplement: Supplementary file 1 [file children-12-01533-s001.zip › children-3929092-supplementary-check list.pdf]

PRISMA 2020 Checklist

Section and Topic	Item #	Checklist item	Location where item is reported
**TITLE**			
Title	1	Timing of Orthodontic Intervention for Pediatric Class II Malocclusion: A Systematic Review on Early vs Late Treatment Outcomes	Lines 2-3
**ABSTRACT**			
Abstract	2	Background/Objectives: The optimal timing for orthodontic treatment in pediatric patients with malocclusion, particularly Class II discrepancies, remains a topic of ongoing clinical debate. Early treatment during the mixed dentition stage can utilize craniofacial growth, while late intervention may be more effective. This systematic review aimed to compare outcomes of early versus late orthodontic treatment to assess their relative effectiveness. Methods: A systematic review was conducted in accordance with PRISMA guidelines, encompassing randomized controlled trials and observational studies from 2015 to 2025. Eleven studies comparing early and late treatment groups were included. The risk of bias was evaluated using the RoB 2.0 tool. Statistical analysis was performed using Python 3.12, with Mann–Whitney U tests and effect size calculations to identify differences between groups. Results: Of the eleven studies, eight reported statistically significant improvements favoring early orthodontic intervention. Early treatment groups showed greater increases in maxillary and mandibular arch dimensions. Notable skeletal effects included a reduction in gonial angle ($p < 0.01$), improved anteroposterior jaw relationships, and increases in airway dimensions, such as rl1–rl2 ($p = 0.010$). Studies utilizing headgear and growth-modifying appliances reported earlier and more favorable eruption patterns and alignment. Effect sizes were moderate to large, supporting the clinical importance of early intervention. Conclusions: Early orthodontic intervention provides measurable benefits in skeletal development, dental arch expansion, and treatment efficiency. While late treatment may be suitable for some cases, personalized planning remains crucial. Additional large-scale, standardized longitudinal studies are necessary to refine timing protocols in pediatric orthodontics.	Lines 51-72
**INTRODUCTION**			
Rationale	3	Although malocclusion is highly prevalent in children, the optimal timing of orthodontic intervention—early versus late—remains debated, with inconsistent evidence on outcomes, burden of care, and long-term stability.	Lines 77-122
Objectives	4	This review aims to compare the clinical outcomes of early versus late orthodontic intervention for pediatric malocclusion, specifically evaluating skeletal, dental, and airway-related changes, the effectiveness of growth-modifying appliances at different developmental stages, the impact on treatment needs such as extractions or fixed appliances, and overall effectiveness, stability, and efficiency.	Lines 123-140
**METHODS**			
Eligibility criteria	5	Studies were included if they compared early (ages 6–9) versus late (ages 10–14) orthodontic interventions in children with malocclusion, used fixed, removable, or functional appliances, and reported outcomes such as occlusal changes, treatment duration, stability, or patient-centered measures; exclusions applied to studies involving craniofacial syndromes/clefts, non-English publications, or designs lacking robust methodology, and eligible studies were grouped by intervention timing (early vs. late) for synthesis.	Lines 153-176
Information sources	6	PubMed, Google Scholar	Lines180-182
Search strategy	7	The electronic search strategy was conducted in PubMed and Google Scholar, covering the period 2014–2024. Search terms combined controlled vocabulary and free-text words related to timing of orthodontic treatment, pediatric malocclusion, and specific appliances (e.g., 'early orthodontic treatment,' 'late orthodontic intervention,' 'Class II malocclusion,' 'cervical headgear,' 'eruption guidance appliance,' 'modified C-palatal plate'). Filters were applied to restrict the studies to those involving human subjects, pediatric populations aged 6–14 years, and articles published in English. Additional limits included study designs (randomized controlled trials, controlled clinical trials, cohort studies, and retrospective studies) and the availability of full-text articles. A total of 496 articles were identified, duplicates (n=64) and unrelated records (n=358) were excluded, leaving 51 full-text articles assessed for eligibility, of which 11 met inclusion criteria for qualitative synthesis.	Lines 153-175
Selection process	8	All identified records were screened in a two-stage process: titles/abstracts were followed by a full-text review. Two reviewers independently assessed each record for eligibility against predefined inclusion and exclusion criteria. Discrepancies were resolved through discussion or by consulting a third reviewer. Automation tools (ElicitAI) were used in the initial phase to support identification and deduplication, but all final inclusion decisions were made manually by the review team.	Lines 153-175
Data collection	9	Two reviewers independently verify extracted data from each included study using a standardized form.	Lines 153-175

Section and Topic	Item #	Checklist item	Location where item is reported
process			
Data items	10a	Skeletal changes – cephalometric measurements of mandibular and maxillary growth.<br>Dental changes – overjet, overbite, arch length, and occlusal relationships.<br>Airway-related changes – upper airway dimensions and functional outcomes.<br>Treatment duration – length of active orthodontic therapy.<br>Post-treatment stability and relapse – maintenance or loss of treatment effects over time.<br>Patient-centered outcomes – satisfaction, psychosocial impact, and quality of life.	Table 1
	10b	Participant characteristics – age range (6–14 years), sex distribution, baseline malocclusion type/class, and sample size.<br>Intervention characteristics – type of appliance (fixed, removable, functional), timing of treatment (early vs. late), treatment protocol, and follow-up duration.<br>Comparator characteristics – control or comparison group details (e.g., delayed treatment, alternative appliance).<br>Study characteristics – study design (RCT, CCT, cohort, retrospective), country/setting, year of publication.<br>Risk of bias factors – randomization, blinding, drop-out rates, and reporting transparency.	Table 1
Study risk of bias assessment	11	The risk of bias was independently assessed by two reviewers using the Cochrane RoB 2 tool for randomized trials and the ROBINS-I tool for non-randomized studies, with disagreements resolved by consensus.	Lines 233-237
Effect measures	12	Skeletal changes – mean differences in cephalometric measurements (e.g., ANB angle, mandibular length).<br>Dental changes – mean differences in overjet, overbite, arch length, and occlusal scores.<br>Airway-related changes – mean differences in upper airway dimensions (mm).<br>Treatment duration – mean difference in months of active treatment.<br>Post-treatment stability/relapse – risk ratios or proportions of relapse, where reported.<br>Patient-centered outcomes – standardized mean differences in validated psychosocial or quality-of-life scores.	Table 1
Synthesis methods	13a	Studies were tabulated by intervention type, timing (early vs. late), and reported outcomes, and then compared against the predefined eligibility criteria to determine inclusion in each synthesis.	Table 1
	13b	Data were extracted as reported; when summary statistics were missing or presented in non-standard formats, values were converted to consistent units (e.g., millimeters, months), and incomplete information was noted as 'not reported' without imputation.	Table 1
	13c	Results of individual studies were tabulated in evidence tables summarizing study characteristics and outcomes.	Table 1
	13d	A synthesis was conducted due to heterogeneity in study designs, interventions, and outcome measures. The results were grouped by treatment timing (early vs. late) and outcome domain (skeletal, dental, airway, stability, and patient-centered). No meta-analysis was performed, as pooling was not feasible.	Table 1
	13e	Not applicable.	-
	13f	Not applicable.	-
Reporting bias assessment	14	The risk of reporting bias was assessed qualitatively by comparing reported outcomes with the study protocols or methods sections, where available.	Lines 233-237
Certainty assessment	15	The certainty of evidence for each outcome was assessed using the RoB 2 approach, considering the risk of bias, inconsistency, indirectness, imprecision, and publication bias; overall confidence ratings were assigned as low risk, some concerns, or high risk of bias per study/outcome.	Lines 233-237

PRISMA 2020 Checklist

Section and Topic	Item #	Checklist item	Location where item is reported
**RESULTS**			
Study selection	16a	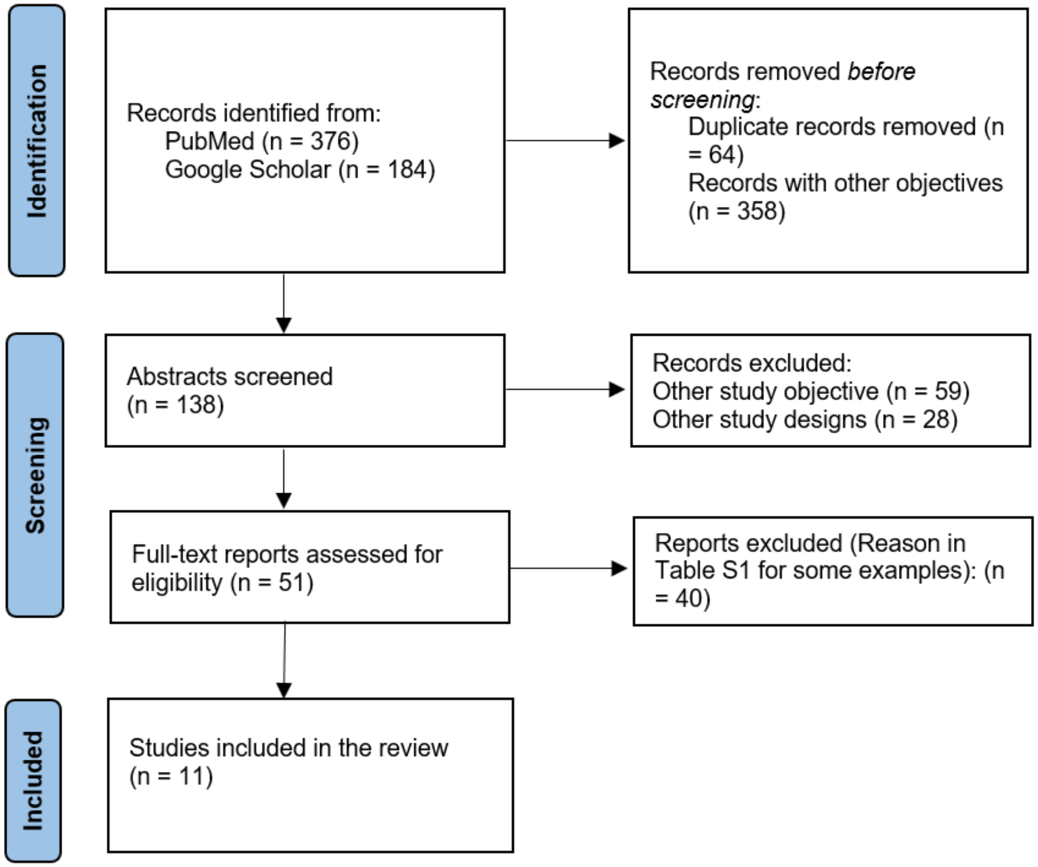	Figure 1
	16b	(table below)	Supplementary materials Table S1

Study ID	Title	Reason for exclusion
Yan S. et al., 2025	A study on the extraoral cervical traction in the treatment of skeletal Class II division 1 malocclusion in mixed dentition	Wrong outcome, old study
Martin C. et al., 2018	Discontinuation of Orthodontic Treatment: What are the early predictive factors?	Wrong study design
Tzemach M. et al., 2014	Early orthodontic treatment for growth modification by functional appliances--pros and cons	Wrong study design
Li W.R. et al., 2022	Early orthodontic treatment in the early permanent dentition	Wrong outcome
Matthews-Brzozowska T., 2015	Fixed appliance therapy in patients with impaired short-circuit in the anterior part of the maxilla	Wrong outcome

Section and Topic	Item #	Checklist item	Location where item is reported

El-Medawar L. et al., 2005	Orthopedic aspects of Distal Active Concept therapy applied in the mixed dentition. Comparative study with an untreated population	Wrong outcome, old study
Zaidan L., 2022	A Comparison of Treatment Success between Functional and Camouflage Orthodontic Treatments in cl II Malocclusion	Wrong outcome
Nichols G. et al. 2024	Are changes in malocclusion associated with adulthood psychosocial well-being?	Wrong outcome
Kalha A et al., 2023	Early Orthodontic Treatment Reduced Incisal Trauma in Children with Class II Malocclusions	Wrong study design
Thiruvenkatachari R.B. et al., 2018	Early Orthodontic Treatment for Class II Malocclusion Reduces the Chance of Incisal Trauma: Results of a Cochrane Systematic Review	Wrong study design

Section and Topic	Item #	Checklist item	Location where item is reported
Study characteristics	17	Julku et al., 2019[9] (Finland, RCT, n=56) – Class II; cervical headgear; skeletal and airway cephalometrics; ~11 yrs duration; 4.2 yrs follow-up.<br>Käsmä et al., 2025[12] (Finland, RCT, n=67) – Class II; cervical headgear; eruption timing of canines/molars; ~14 yrs duration.<br>Kallunki et al., 2021[13] (Sweden, RCT, n=56) – Class II with excessive overjet; headgear activator; cost-effectiveness; 2 yrs.<br>Julku et al., 2019[10] (Finland, RCT, n=67) – Class II; cervical headgear; skeletal/dental cephalometrics; 26–24 months treatment.<br>Kim et al., 2024[14] (Korea, RCT, n=71) – Hyperdivergent Class II; modified C-palatal plates vs. headgear; cephalometric vertical control.<br>Männchen et al., 2022[17] (Italy, Retrospective, n=527) – Class II; headgear, activators, space maintainers vs. fixed appliances; cephalometric & extraction needs; 6 yrs.<br>Mandall et al., 2022[15] (UK, RCT, n=75) – Class II; RME + facemask; cephalometric, overjet, psychosocial; ≥3 yrs.<br>Julku et al., 2018[11] (Finland, RCT, n=67) – Class II; cervical headgear; skeletal and airway cephalometrics; 11 yrs duration; 4.5 yrs follow-up.<br>Myrlund et al., 2018[19] (Norway, Prospective cohort, n=35) – Class II; Eruption Guidance Appliance; overjet, crowding; ~5–6 yrs.<br>Fourneron et al., 2020[18] (France, Retrospective, n=40) – Unilateral posterior crossbite; Quad Helix expansion; mandibular asymmetry; 18 months.<br>Hannula et al., 2023[16] (Finland, RCT, n=46) – Class II; cervical headgear; dental arch dimensions; ~14 yrs duration; 10.4 yrs follow-up.	Table 1
Risk of bias in studies	18	Julku et al., 2019[9] (RCT) – Low risk overall; some concerns with blinding.<br>Käsmä et al., 2025[12] (RCT) – Low risk; robust randomization, limited reporting on allocation concealment.<br>Kallunki et al., 2021[13] (RCT) – Low risk; complete outcome reporting, but potential performance bias.<br>Julku et al., 2019 (RCT)[10] – Low risk; similar concerns with blinding.<br>Kim et al., 2024 (RCT)[14] – Low risk; some concerns about attrition.<br>Männchen et al., 2022[17] (Retrospective) – Moderate risk; confounding and selection bias.<br>Mandall et al., 2022[15] (RCT) – Low risk; minor concerns on allocation concealment.<br>Julku et al., 2018[11] (RCT) – Low risk; incomplete blinding reporting.	Supplementary materials Table S2

PRISMA 2020 Checklist

Section and Topic	Item #	Checklist item							Location where item is reported
		Myrlund et al., 2018[19] (Cohort) – Moderate risk; potential selection and reporting bias.<br>Fourneron et al., 2020[18] (Retrospective) – Moderate risk; confounding and incomplete follow-up.<br>Hannula et al., 2023[16] (RCT) – Low risk; complete outcome reporting, minor concerns with attrition.							
Results of individual studies	19	Julku et al., 2019 (EJO) [9]	Skeletal (gonial angle), AP relationship	Early CH vs. Late CH	NR	Greater gonial angle reduction in Early vs. Late	$p < 0.01$	Also improved anteroposterior jaw relationship in both groups; no clinically large differences reported.	Table 1
		Käsmä et al., 2025 (EJO) [12]	Eruption timing & alignment	Early CH vs. Late CH	NR	Later (LG) had earlier eruption of 2nd molars and canines with better alignment; EG had more distal tipping & 2nd–3rd molar overlap	All results $p < 0.05$	Follow-up across T0–T4 ~10.4 yrs.	
		Kallunki et al., 2021 (EJO) [13]	Costs; clinical effects	Mixed vs late mixed dentition	NR	No significant cost differences or major clinical differences between timings	NS	2-year RCT; excessive overjet sample.	
		Julku et al., 2019 (EJO) [10]	Dental arch dimensions	Early CH vs. Late CH	NR; male subgroup effects	Early > Late for arch length & transverse gains (notably in males)	Significant (values NR)	EG males > LG males for upper/lower molar width & mandibular arch length.	
		Kim et al., 2024 (Angle) [14]	Vertical control (SN-GoGn, FMA)	Early MCPP vs. Late MCPP; Early HG vs. Late HG	SN-GoGn change: Early MCPP −0.6°, Early HG +1.7°	Early MCPP showed greater vertical control than Late MCPP and HG	P < .01 for key comparisons	Hyperdivergent Class II adolescents.	
		Männchen et al., 2022	Treatment burden & outcomes	Early mixed protocols vs.	NR	EG reduced extraction rate by	NR	Total treatment time ↑ in EG but FFA duration ↓.	

PRISMA 2020 Checklist

Section and Topic	Item #	Checklist item	Location where item is reported

(Children) [17]		Late fixed protocols		22.2%; 15.9% fewer needed full fixed appliances; >5° less incisor proclination (non-extraction cases)		
Mandall et al., 2022 (J Orthod) [15]	Skeletal/dental & psychosocial	Immediate vs 18-month delayed (Twin Block context w/ RME+FM)	ANB −1.6° (95% CI 0.89–2.29); Overjet −5.06 mm (95% CI 3.96–6.16)	Immediate treatment improved ANB and overjet more by DC2	p < 0.001 (ANB, overjet); treatment time p = 0.004	No significant differences in psychological outcomes.
Julku et al., 2018 (EJO) [11]	Airway & skeletal	Early CH vs. Late CH	NR	Early > Late for retroglossal airway (rl1–rl2) increase; posterior maxillary movement in early males (SNA ↓)	rl1–rl2 p = 0.010; SNA p < 0.001 (T0–T1), p = 0.012 (T0–T2)	Sex-specific correlations significant (p < 0.001).
Myrlund et al., 2018 (Angle) [19]	Dentoalveolar outcomes	Pre vs post EGA; follow-up	NR	Significant improvements in overjet, overbite, sagittal molar relation, mandibular crowding	Significant (values NR)	Retention compliance linked to smaller overjet/overbite.
Fourneron et al., 2020 (Int Orthod) [18]	Mandibular asymmetry (ΔL, ΔH)	Early (<7y) vs. Late (≤13y) QH expansion	NR	Early group showed +1.0 mm greater correction in corpus asymmetry	p = 0.008	12-month follow-up.
Hannula et al., 2023 (EJO) [16]	Arch dimensions (3D scans)	Early CH vs. Late CH	NR	Early > Late for maxillary arch length, intermolar width, upper canine width,	P-values: .048; .002; .001; .035 respectively	Longitudinal T0–T4 follow-up to age ~17.7 years.

Section and Topic	Item #	Checklist item	Location where item is reported
		and U5–U6 distance	
Results of syntheses	20a	Skeletal outcomes:<br>Contributing studies (Julku 2018, Julku 2019, Kim 2024, Hannula 2023) were predominantly RCTs with low overall risk of bias, though some lacked detail on blinding. Findings consistently indicated modest skeletal benefits from early treatment, with effect sizes significant in cephalometric parameters (e.g., gonial angle, ANB).<br>Dental and occlusal outcomes:<br>Evidence came from RCTs and cohort studies (Julku 2019, Kallunki 2021, Hannula 2023, Myrlund 2018). Risk of bias was generally low to moderate; prospective cohorts had potential selection bias. Early treatment improved overjet, arch length, and occlusal relationships, though late treatment sometimes yielded more stable eruption patterns.<br>Airway outcomes:<br>Two RCTs (Julku 2018, Julku 2019) provided cephalometric airway measures. Both were low risk of bias and suggested early treatment increased airway dimensions in males, with significant skeletal–airway correlations.<br>Treatment burden and efficiency:<br>Contributing evidence (Männchen 2022, Mandall 2022, Kallunki 2021) included retrospective and RCT designs. Risk of bias was low in the RCTs, moderate in the retrospective. Results were mixed: early treatment reduced need for extractions and appliances but often lengthened total treatment time.<br>Patient-centered outcomes:<br>Only Mandall 2022 (RCT) explicitly measured psychosocial impact. Risk of bias was low, but no significant differences were detected between early and late groups.	Lines 267-305
	20b	Comparative direction overall (early vs. late): Across the included studies, early treatment was more frequently associated with favorable outcomes than late treatment (Mann–Whitney $U = 8.0$, $p = 0.0067$), indicating a direction of effect favoring early intervention.<br>Baseline comparability across studies: The mean age at treatment start did not differ between the early/both and late/both study groupings ($t = -0.10$, $p = 0.92$); follow-up duration was also comparable ($t = -0.01$, $p = 0.99$).<br>Protocol complexity (single vs. mixed modalities): Estimated success scores were higher with single-modality protocols (mean 2.75 ± 0.46) than mixed protocols (2.33 ± 0.58); difference not statistically significant (Mann–Whitney $U = 17.0$, $p = 0.27$), with a small to moderate effect size ($r = 0.31$) favoring single-modality approaches.	Lines 336-338
	20c	Heterogeneity was explored by study design, baseline age, follow-up duration, appliance modality, and skeletal pattern. No significant differences were found for age or follow-up, while qualitative variation was linked to appliance type, sex-specific growth responses, and outcome measurement methods.	Lines 336-338
	20d	Not applicable	-
Reporting biases	21	No formal statistical assessment of reporting bias was performed due to the small number of studies per outcome.	-
Certainty of evidence	22	For skeletal outcomes, certainty was rated moderate due to consistent findings across low-risk RCTs but limited precision. Dental/occlusal outcomes were judged to be low to moderate, downgraded due to heterogeneity and selective reporting. Airway outcomes were of low certainty, as evidence came from a small number of trials with sex-specific effects and limited generalizability. Treatment burden/efficiency evidence was of low certainty, reflecting variability between retrospective and prospective designs. Patient-centered outcomes were rated very low, based on a single RCT with limited statistical power.	Linse 309-329
**DISCUSSION**			
Discussion	23a	This review suggests that early orthodontic intervention can provide advantages in reducing overjet, improving dental arch dimensions, and	Lines 409-502

PRISMA 2020 Checklist

Section and Topic	Item #	Checklist item	Location where item is reported
		mitigating airway restrictions. In contrast, late intervention during the pubertal growth spurt often yields more stable skeletal corrections and benefits in eruption timing. These findings align with prior reviews highlighting that early treatment may reduce trauma risk and psychosocial burden. Still, they also reinforce concerns about extended treatment duration and the frequent need for a second treatment phase. In the context of existing evidence, our results support an individualized approach: early treatment may be justified for high-risk malocclusion cases, whereas late treatment remains preferable for skeletal Class II discrepancies requiring growth modification.	
	23b	The included evidence was limited by heterogeneity in study designs, appliances, and outcome measures, with several studies lacking standardized reporting of summary statistics or confidence intervals. Many trials had small sample sizes, and long-term follow-up was inconsistently reported. Additionally, observational studies carry risks of selection bias and confounding. Patient-centered outcomes were rarely assessed, which reduced the certainty of conclusions regarding quality of life. These limitations constrain the strength and generalizability of the findings.	Lines 516-528
	23c	Restrictions may limit the review process to English-language publications and databases (PubMed, Semantic Scholar, Google Scholar), which could introduce language or publication bias. Data extraction was performed independently by two reviewers; however, no contact was made with the study authors to clarify missing or unclear information, which may have affected the completeness of the data.	-
	23d	Discuss implications of the results for practice, policy, and future research.	
**OTHER INFORMATION**			
Registration and protocol	24a	PROSPERO database under the ID number CRD420251157567	-
	24b	https://www.crd.york.ac.uk/PROSPERO/view/CRD420251157567	-
	24c	No modifications have been made.	-
Support	25	We acknowledge the Victor Babes University of Medicine and Pharmacy, Timisoara, Romania, for covering the publication costs for this research paper.	Lines 551-552
Competing interests	26	The authors declare no conflicts of interest	Line 557
Availability of data, code and other materials	27	All data extracted from the included studies, the analytic dataset, and the Python code used for statistical analyses are available in the supplementary materials of this article. The template data collection form and evidence tables are provided as appendices. No additional unpublished materials were generated.	-

From: Page MJ, McKenzie JE, Bossuyt PM, Boutron I, Hoffmann TC, Mulrow CD et al. The PRISMA 2020 statement: an updated guideline for reporting systematic reviews. BMJ 2021;372:n71. doi: 10.1136/bmj.n71.
This work is licensed under CC BY 4.0. To view a copy of this license, visit https://creativecommons.org/licenses/by/4.0/
