# Supplementary material for: Timing of Orthodontic Intervention for Pediatric Class II Malocclusion: A Systematic Review on Early vs. Late Treatment Outcomes
Source: Children (Basel). 2025 Nov 13;12(11):1533. doi: 10.3390/children12111533 (PMC12651552; doi:10.3390/children12111533)
Supplement: Supplementary file 1 [file children-12-01533-s001.zip › children-3929092-supplementary.pdf]

children MDPI

Supplementary Materials

Table S1. Excluded Studies and Reason for Exclusion.

Study ID	Title	Reason for exclusion
Yan S. et al., 2025	A study on the extraoral cervical traction in the treatment of skeletal Class II division 1 malocclusion in mixed dentition	Wrong outcome, old study
Martin C. et al., 2018	Discontinuation of Orthodontic Treatment: What are the early predictive factors?	Wrong study design
Tzemach M. et al., 2014	Early orthodontic treatment for growth modification by functional appliances--pros and cons	Wrong study design
Li W.R. et al., 2022	Early orthodontic treatment in the early permanent dentition	Wrong outcome
Matthews-Brzozowska T., 2015	Fixed appliance therapy in patients with impaired short-circuit in the anterior part of the maxilla	Wrong outcome
El-Medawar L. et al., 2005	Orthopedic aspects of Distal Active Concept therapy applied in the mixed dentition. Comparative study with an untreated population	Wrong outcome, old study
Zaidan L., 2022	A Comparison of Treatment Success between Functional and Camouflage Orthodontic Treatments in cl II Malocclusion	Wrong outcome
Nichols G. et al. 2024	Are changes in malocclusion associated with adulthood psychosocial well-being?	Wrong outcome
Kalha A,. et al., 2023	Early Orthodontic Treatment Reduced Incisal Trauma in Children with Class II Malocclusions	Wrong study design
Thiruvenkatachari R.B. et al., 2018	Early Orthodontic Treatment for Class II Malocclusion Reduces the Chance of Incisal Trauma: Results of a Cochrane Systematic Review	Wrong study design

Table S2. RoB 2.0 Risk of Bias Assessment

Study ID	Randomization	Deviation from Intervention	Missing Outcome	Measurement of Outcome	Selective Reporting	Overall Risk
Julku et al., 2019[9]	Low	Low	Low	Low	Low	Low
Käsmä et al., 2025[12]	Low	Low	Low	Low	Low	Low
Kallunki et al., 2021[13]	Some concerns	Low	Low	Low	Low	Low
Julku et al., 2019[10]	Low	Low	Low	Low	Low	Low
Kim et al., 2024[14]	Some concerns	Some concerns	Low	Low	Low	Some concerns
Männchen et al., 2024[17]	Some concerns	Low	Moderate	Moderate	Moderate	Moderate
Mandall et al., 2022[15]	Low	Low	Low	Low	Low	Low
Julku et al., 2018[11]	Low	Low	Low	Low	Low	Low
Myrlund et al., 2018[19]	Low	Low	Low	Low	Low	Low

Fourneron et al., 2020[18]	Some concerns	Low	Low	Low	Low	Some concerns
Hannula et al., 2023[16]	Low	Low	Low	Low	Low	Low

Table S3. Study characteristics (extended)

Study (Year)	Treatment Details	Time Points / Measurements	Variables Assessed	Analytical Methods	Additional Notes
Julku et al., 2019 [9]	Kloehn-type CH, 500 g force, 8–10 h/night	T0 (baseline), T1 (start), T2 (end)	Cephalometric skeletal & airway dimensions (N-ANS, NSL-PL, gonial angle)	WinCeph 8.0	Gender-specific cephalometric differences; NSL-PL ↑ is increased in both groups.
Käsmä et al., 2025 [12]	CH treatment	T0–T4 (7.3–17.8 yrs)	Eruption timing, molar inclination, overlap	DPT radiographs	LG showed better eruption and less molar overlap.
Kallunki et al., 2021 [13]	Headgear activator	Pre–post over 2 yrs	Treatment costs, skeletal/lip closure	Economic analysis	No cost or effectiveness differences between groups.
Julku et al., 2019 [10]	Kloehn-type CH	EG: Treated from T0 to T1 (26 months), starting at a mean age of 7.8<br>LG: Treated from T1 to T2 (24 months), starting at a mean age of 9.5	Males benefited more from early CH treatment, with broader, longer arches and spontaneous lower arch expansion	WinCeph 8.0	EG showed significant maxillary arch length and transverse gains at T0–T1<br>At T2, EG males had greater transverse width (upper/lower first molars) and longer mandibular arches than LG males
Kim et al., 2024 [14]	MCPP vs. CH	Early (9.9) vs. Late (12.3)	FMA, SN-GoGn, vertical control	Lateral cephalograms	Early MCPP ↓ SN-GoGn; $p < 0.01$.
Männchen et al., 2022 [17]	Headgear, Teuscher activators, lip bumpers	T1–T2	ANB, Wits, SN/MeGo, overjet, space balance	Cephalograms & casts	EG ↓ extraction rate by 22%; ↓ incisor proclination >5°.
Mandall et al., 2022	RME + facemask	DC1–DC3 (0–3 yrs)	ANB, overjet, psychosocial scores	Cephalograms, CPQ11–14, OASIS	ANB ↓ 1.6°, overjet ↓ 5 mm; $p < 0.001$.
Julku et al., 2018 [11]	Kloehn-type CH	T0–T2 (11 yrs)	SNA, palatal angle, airway dimensions (rl1–rl2, va1–va2)	WinCeph 8.0	Early males: ↑ airway; $p < 0.010$. (SNA ↓; $p < 0.001$ at T0–T1, $p = 0.012$ at T0–T2)<br>palatal line–mandibular line angle ↓ in early treatment females ($p = 0.018$) and males ($p = 0.037$) at T0–T1

					Retroglossal airway (rl1–rl2) ↑ in early treatment males at T0–T1 (p = 0.010) Positive correlation between skeletal and airway changes in early treatment males (p < 0.001) rl1–rl2 airway diameter ↑ in early vs. late treatment males at T0–T1 (p = 0.010) va1 ‑ va2 distance ↑ in early vs. late group at T0–T1 (p = 0.030)
Myrlund et al., 2018 [19]	EGA	Pre, post, 4–5 yrs	Overjet, overbite, crowding	Dental casts	Long-term improvement is linked to compliance.
Fourneron et al., 2020 [18]	Quad Helix	12 mo	Mandibular corpus length ΔL, ramus height ΔH	Radiography	Corpus asymmetry correction +1 mm (p = 0.008).
Hannula et al., 2023 [16]	CH	T0–T4 (7.3–17.7 yrs)	Arch length/width (U1–U9, L1–L6)	3Shape Ortho Analyzer™	EG > LG in multiple arch parameters (p < 0.05).

Table S4. Extracted data for analyze

Study ID	Country	Sample Size	Intervention type	Outcome	p-value
Julku et al., 2019[9]	Finland	56	Kloehn-type cervical headgear (CH)	Cephalometric changes (skeletal and airway)	< 0.05, < 0.01
Käsmä et al., 2025[12]	Finland	67	CH treatment	Tooth eruption timing, inclination, and molar overlap	< 0.05
Kallunki et al., 2021[13]	Sweden	56	Headgear activator treatment	Treatment costs, overjet, and molar relation	N/A
Julku et al., 2019[10]	Finland	67	Kloehn-type CH with a long outer bow	Maxillary/mandibular arch changes	< 0.05
Kim et al., 2024[14]	Korea	71	Modified C-palatal plates (MCPP) and CH	Mandibular plane angle, FMA, SN-GoGn	< 0.01

Männchen et al., 2024[17]	Italy	527	EG: Headgear, growth appliances, Teuscher activators, and space maintainers (lingual arch, lip bumper, utility arch) LG: Full fixed appliances with elastics or noncompliance devices, plus headgear and growth appliances	Extraction rate, FFA need, incisor proclination	<0.05
Mandall et al., 2022[15]	United Kingdon	75	Rapid maxillary expansion using a bonded acrylic-splint expander and facemask therapy	ANB, overjet, treatment time	< 0.001, 0.004, > 0.05
Julku et al., 2018[11]	Finland	67	Kloehn-type CH	Cephalometric and airway changes	< 0.001, 0.012, 0.018, 0.037, 0.010, 0.030
Myrlund et al., 2018[19]	Norway	35	Eruption Guidance Appliance (EGA)	Overjet, overbite, molar relation, crowding	N/A
Fourneron et al., 2020[18]	France	40	Quad Helix (QH) for maxillary expansion	Mandibular corpus asymmetry	0.008
Hannula et al., 2023[16]	Finland	46	CH	Dental arch dimensions	0.048, 0.002, 0.031, 0.035, 0.001
